# Proinflammatory Extracellular Vesicle-Mediated Signaling Contributes to the Induction of Neuroinflammation in Animal Models of Endotoxemia and Peripheral Surgical Stress

**DOI:** 10.1007/s10571-020-00905-3

**Published:** 2020-06-18

**Authors:** F. Fricke, J. Gebert, J. Kopitz, K. Plaschke

**Affiliations:** 1 Institute of Pathology, University Hospital Heidelberg, Im Neuenheimer Feld 224, 69120 Heidelberg, Germany; 2 Department of Anesthesiology, University Hospital Heidelberg, Im Neuenheimer Feld 110, 69120 Heidelberg, Germany

## Abstract

Peripheral inflammation induced by endotoxemia or surgical stress induces neuroinflammation thereby causing neurological symptoms ranging from sickness behavior to delirium. Thus, proinflammatory signaling must be operative between the periphery and the central nervous system (CNS). In the present study, we tested whether nanometer-sized extracellular vesicles (EVs) that were produced during the peripheral inflammatory process have the capacity to induce neuroinflammation. Conditions of endotoxemia or surgical intervention were simulated in rats by lipopolysaccharide (LPS) injection or partial hepatectomy (HpX). EVs were concentrated from these animals and tested for their proinflammatory action (I) in a microglial cell line and (II) by intracerebroventricular and (III) by intravenous injections into healthy rats. EVs from both conditions induced the secretion of cytokines from the glial cell line. Intracerebroventricular injection of the EVs caused the release of inflammatory cytokines to the cerebrospinal fluid indicating their pro-neuroinflammatory capacity. Finally, proinflammatory EVs were shown to pass the blood–brain barrier and induce neuroinflammation after their intravenous injection. Based on these data, we suggest that EV-associated proinflammatory signaling contributes to the induction of neuroinflammation in endotoxemia and peripheral surgical stress. Preliminary results suggest that peripheral cholinergic signals might be involved in the control of proinflammatory EV-mediated signaling from the periphery to the brain.

## Introduction

Systemic inflammation, e.g., elicited by bacterial infection, large injuries or surgeries, induces physiological and neurological changes that are commonly referred to as ‘sickness behavior’ which is characterized by a decline of cognitive function, somnolescence, fever, decreased food intake and general fatigue. Since it is now quite clear that systemic inflammation can cause brain inflammation, such neuroinflammation is considered the main cause of the neurological changes ranging from sickness behavior to delirium (Poon et al. 2015). Consequently, the brain must be able to sense peripheral inflammation by communicating with the innate immune system. Several pathways of how the brain senses a peripheral inflammation have been suggested. For example, locally produced cytokines may activate primary afferent nerves such as the vagal nerves (Bluthe et al. 1994) thereby triggering signaling to the CNS (Chiu et al. 2013; Goehler et al. 2000). Also, circumventricular organs (CVO) are cerebral areas with incomplete endothelial blood–brain barrier (BBB) and therefore regarded as "gates to the brain". Thus, peripherally released proinflammatory cytokines may enter the CNS through this gate and elicit neuroinflammation (Wuerfel et al. 2010). Moreover, peripheral cytokines can gain access to the brain through specific transport systems in the BBB (Banks 2006). Finally, inflammatory damage to the microvascular endothelial cell monolayer that constitutes the luminal BBB surface, can lead to elevated BBB permeability which opens an access path for proinflammatory signaling molecules to the CNS (Rochfort and Cummins 2015).

**Fig. 1:**
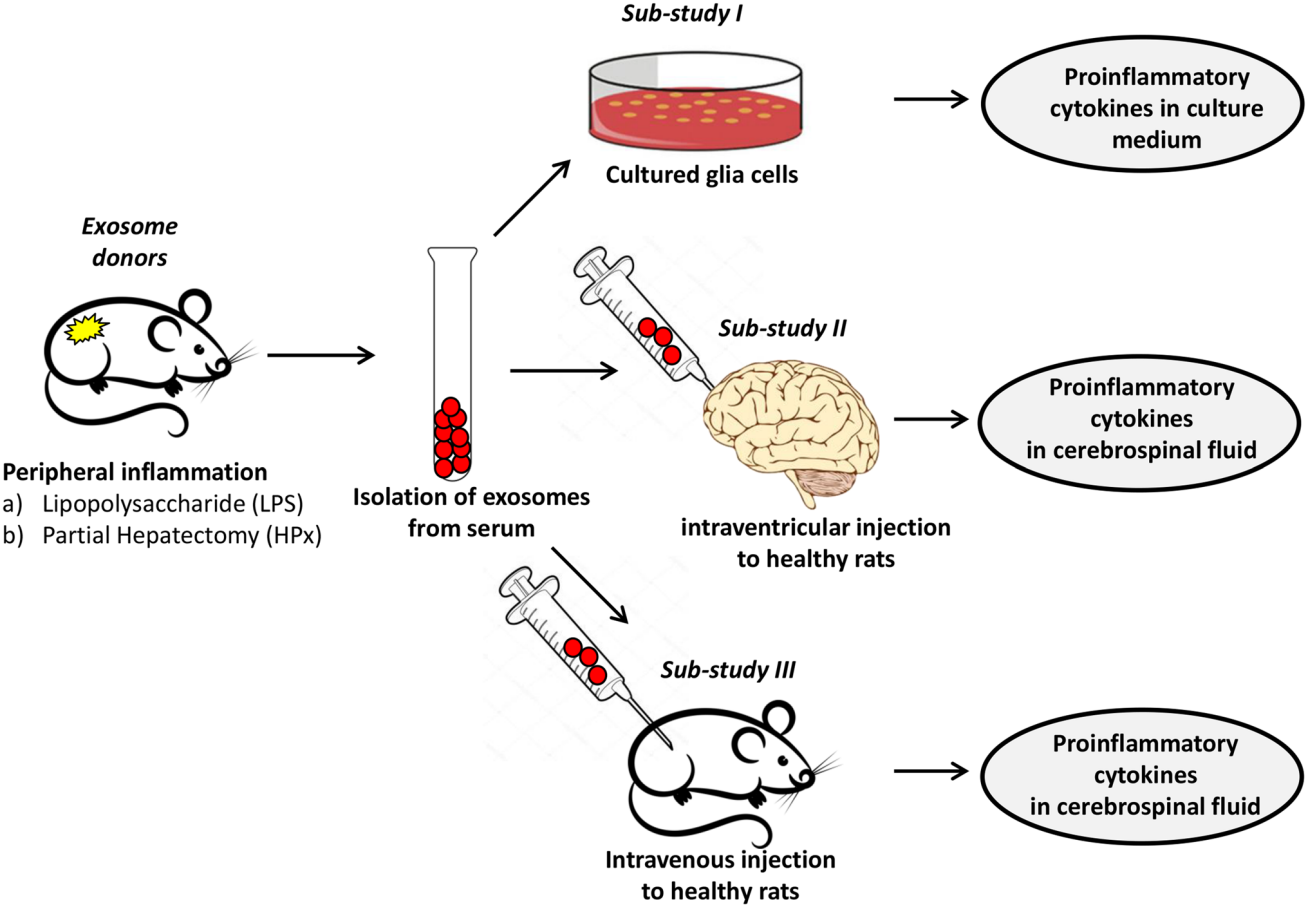
Experimental design. Animals were treated either with LPS or by HpX. Blood was sampled and EVs were isolated 4 h after LPS injection or 2 h after HpX. EVs from untreated animals were taken as normal controls. *Sub-study I:* concentrated EVs were added to cultured glia cells and inflammatory cytokines assayed in the conditioned medium was assayed after 1 h. *Sub-study II*: EVs were administered by an intracerebroventricular (icv) injection to healthy rats. CSF was taken 1 h after the injection and assayed for inflammatory cytokines. *Sub-study III:* EVs were administered by an intravenous (i.v.) injection via the femoral vein to healthy rats

Recently a novel mechanism for peripheral signaling to the CNS has been suggested. Based on the observation that extracellular vesicles are capable of passing the BBB, nominated them as potential vehicles to transfer peripheral signaling molecules to the brain (Chen et al. 2016; Matsumoto et al. 2017). The generic term “Extracellular Vesicles” (EVs) describes a heterogeneous population of lipid bilayer-enclosed particles that are secreted by nearly all cells into the extracellular space (Becker et al. 2016; Mathieu et al. 2019; Witwer and Théry 2019). EVs can be classified into different EV subtypes according to their biogenesis and sizes. The two most prominent subclasses are exosomes (30 to 150 nm) originating from the endosomal pathway and microvesicles (100 to 1000 nm) that are plasma membrane-derived (Stahl and Raposo 2018). Circulating EVs are considered key players in cell-to-cell communication by transferring a molecular message encoded by their cargo to recipient cells (Raposo and Stahl 2019; van Niel et al. 2018; Wortzel et al. 2019). EVs are found in bodily fluids, such as blood, cerebrospinal fluid (CSF), saliva, and urine. Their cargo consists of nucleic acids, proteins, lipids and carbohydrates (van Niel et al. 2018). EVs present in the CSF have already been shown to be involved in signal transduction not only among neural cells but also among hematopoietic cells and in the peripheral nervous system (Kawahara and Hanayama 2018). Likewise, EVs in the brain play a role in CNS diseases, such as stroke, Alzheimer's disease, Parkinson's disease, prion disease, and traumatic encephalopathy, with both positive and negative effects (Liu et al. 2019). These pathologies of the brain are associated with a neuroinflammatory response. Neuroinflammation is an innate immune response induced by the microglia and astroglia, which respond to a proinflammatory signal by the production of cytokines, chemokines, reactive oxygen species and secondary messengers (Rama Rao and Kielian 2015). Various recent studies indicate that EVs may play a pivotal role in the initiation and control of neuroinflammation (Gupta and Pulliam 2014; Pascual et al. 2020). Therefore, various approaches have been applied to transfer anti-neuroinflammatory compounds through the BBB to attenuate the neuroinflammatory reactions that are considered to contribute to brain pathologies (Upadhya and Shetty 2019). On the other hand, the transport of proinflammatory EVs released during peripheral inflammation may also reach beyond the BBB and trigger neuroinflammation via glia activation (Li et al. 2018). We addressed this question in two experimental animal models simulating conditions of endotoxemia and surgical stress. The experimental design is outlined in Fig. 1.

## Methods

### Animals

The study was performed on adult male Wistar rats (Janvier Labs from Saint-Berthevin Cedex, France), weighing 200–300 g. They were housed twice per cage in a temperature-controlled room at 22 ± 0.5 °C with a reversed day—night cycle (12 h:12 h, light on at 7 p.m.). Free access to food (LASQc diet, LA Svendi‚ Germany) and water was allowed throughout the experimental period. The experimental protocol was approved by the appropriate review committee of the Medical Faculty of the University of Heidelberg (Germany) and complied with the guidelines of the responsible national government agency (Regierungspraesidium Karlsruhe, Germany, G-104/18) and with international standards.

### Surgical Procedures

Anesthesia was induced with 4 vol% sevoflurane (Abbott, Wiesbaden, Germany) via a vaporizer (Dräger, Lübeck, Germany) and oxygen/nitrous oxide (O_2_:N_2_O = 30:70) and maintained over the entire experimental period with 3.5 vol% sevoflurane and oxygen/nitrous oxide (O_2_:N_2_O = 30:70). After endotracheal intubation with a 16-G catheter, rats were ventilated with 60/min breathing frequency using a rodent respirator (Föhr Medical Instruments, Seeheim-Jugenheim, Germany). Tympanal temperature was monitored and maintained at around 37 °C using a heating pad.

In all animals, catheters were placed in the *arteria* and *vena femoralis sinistra* (5.0 nylon thread) for administering drugs, for drawing blood samples, or for blood gas analysis. Partial hepatectomy (HpX) was conducted as described previously (Plaschke et al. 2018b). Lipopolysaccharide (LPS; 20 mg/kg body weight, from Escherichia coli O111:B4, LOT 028M4022V, Sigma- Aldrich, Munich, Germany) was slowly applied via the catheter in the *vena femoralis sinistra* (Plaschke et al. 2018a). Physostigmine as an inhibitor of the acetylcholine esterase was applied intravenously in a concentration of 0.04 mg/kg as detailed in a previous article (Plaschke et al. 2016).

For intraoperative blood analysis, blood samples (0.1 ml per sampling point) were taken via the *arteria femoralis* at 2 time points: before the surgical procedure was started (T0), and immediately before sacrifice (T1, final). Blood taken with a glass capillary (10 µl) was immediately used to analyze blood [pH value, pCO_2_, pO_2_, bicarbonate (HCO_3_^−^), base excess (BE), hematocrit (Hct), hemoglobin (Hb), potassium (K^+^), sodium (Na^+^), glucose, and lactate concentrations].

After 4 h of LPS or 120 min after HpX, the animals were sacrificed under deep general anesthesia by bleeding out. The coagulated blood was centrifuged within 30 min at 7000 rpm at 20 °C for 15 min and the supernatant was separated and frozen as serum at − 80 °C to assess biochemical parameters or for EV concentration.

### Concentration and Characterization of EVs

#### Concentration

Rat serum samples (250 µl) were thawed at 37 °C and diluted 1:5 [v/v] with PBS (Thermo Fisher Scientific, Waltham, USA). Diluted samples were subjected to differential centrifugation (I. 5000×*g*, 15 min, 4 °C; II. 10,000×*g*, 30 min, 4 °C). Cleared supernatants were transferred to sterile polycarbonate ultracentrifuge tubes and centrifuged at 120,000×*g* (*k-*factor 34.8) for 120 min at 4 °C (TLA-100.2, rotor 100.2, Beckmann-Coulter, Krefeld, Germany). After discarding the supernatant, each EV pellet was resuspended in 20 μl of PBS. EV preparations were stored at − 80 °C.

#### Transmission Electron Microscopy

EV samples were diluted 1:5 [v/v] with PBS and transferred onto 100 mesh formvar-coated copper grids. Negative staining was performed with 2% aqueous uranyl acetate. Air-dried samples were visualized at 80 kV using a JEM 1400 transmission electron microscope (JOEL USA, Peabody, USA) equipped with a Tietz 2 K digital camera.

#### Nanoparticle Tracking

Nanoparticle tracking analysis was performed with the ZetaView PMX110 system and the software 8.04.02 SP2 (Particle Metrix, Inning, Germany) according to the manufacturer's instructions. Measurements were performed at 11 different positions in a dilution of 1:10,000 [v/v] with PBS. Settings for data acquisition were adjusted to a sensitivity of 95%, a shutter of 100, and a frame rate of 30 frames per second.

#### Protein Extraction and Western Blot

Proteins were extracted in RIPA buffer (50 mM Tris–HCl pH 7.4, 150 mM NaCl, 1% Triton X-100, 1% Na-deoxycholate, 0.1% SDS, 0.1 mM CaCl_2_ and 0.01 mM MgCl_2_) supplemented with cOmplete Mini protease inhibitor cocktail inhibitor (Roche, Basel, Switzerland). Protein concentrations were measured by Bradford assay (Bio-Rad Laboratories, Hercules, USA) following the manufacturer's instructions. Per sample, 20 μg (CD63, CD9, Alix) or 40 μg (ApoA1) of protein were separated on 4–20% Bis–Tris gradient gels (Expedeon, Cambridge, UK) and blotted onto a nitrocellulose membrane (Life Technologies, Carlsbad, Germany). After blocking the membrane, the following primary antibodies were used: anti-CD63 (1:300, MX-49.129.5, Santa Cruz, Heidelberg, Germany), anti-CD9 (1:200, C4, Santa Cruz), and goat anti-Alix (1:800, Q19, Santa Cruz), rabbit anti-ApoA1 (1:530, polyclonal ab20453, Abcam). Primary antibodies were diluted in 5% milk/TBST (CD63, CD9, Alix) or in PBS/Tween-20 (ApoA1) and incubated with membranes overnight at 4 °C. The blots were washed with TBST and incubated with a sheep anti-mouse-IgG HRP (1:5000; GE-Healthcare, Chicago, USA), donkey anti-goat-IgG HRP (1:1000; Santa Cruz) or a goat anti-rabbit-IgG HRP (1:2000; Promega, Madison, USA) secondary antibody for 1 h at room temperature. Signals were detected using Western Lightning Plus ECL (Perkin Elmer, Waltham, USA) and a ChemiDoc MP System (Bio-Rad Laboratories, Hercules, USA).

#### Availability of Data and Materials

We have submitted all relevant data of our experiments to the EV-TRACK knowledgebase (EV-TRACK ID: EV200028) (Van Deun et al. 2017).

### Routes of EV Application

#### Cell Culture

Human microglial HMC3 cells (Dello Russo et al. 2018) were obtained from LGC Standards GmbH (Wesel, Germany) and cultured in 96 well plates with DMEM medium containing 10% fetal calf serum (Sigma-Aldrich, Taufkirchen, Germany) and 1% penicillin/streptomycin. At 80% confluency, two concentrations (10 and 50 µg/ml) of EV preparations were added to the medium for 1 h before the medium was used for biochemical assays.

#### Intracerebroventricular (icv) Injection

Rats underwent inhalational anesthesia with 3.5 vol% sevoflurane and 30:70 O_2_:N_2_O and via a rat-adapted mask. 10 µl of a suspension of EVs in sterile 0.9% NaCl (corresponding to 10 µg protein) was injected icv into the brain of healthy rats (*n* = 6). The icv injection procedure was performed as described in detail in a previous study (Plaschke and Hoyer 1993). One hour after icv injection, the cerebral spinal fluid (CSF) was taken (50–200 µl per rat) between the cervical vertebras via a small syringe. In addition, blood was drawn from the femoral vein as described above. Both fluids were immediately stored at − 80 °C before further biochemical analysis.

#### Intravenous (i.v.) Injection

Rats underwent inhalational anesthesia with 3.5 vol% sevoflurane and 30:70 O_2_:N_2_O via a rat-adapted mask. The femoral vein was catheterized. 500 µl of a suspension of EVs (corresponding to 250 µg protein) was injected i.v. into *n* = 6 healthy rats. One hour after i.v. injection, CSF was taken (50–200 µl per rat) between the cervical vertebras via a small syringe. In addition, blood was taken via the femoral vein as described above. Both fluids were immediately stored at − 80 °C before further biochemical analysis.

### Fluorescent Labeling of EVs

Carboxyfluorescein succinimidyl ester (CFSE) was used for sensitive fluorescent labeling of EVs. In particular, EVs (200 µg) were suspended in 100 µl PBS/0.5% BSA. Subsequently, 100 µl of 20 µM CFSE in PBS/0.5% BSA was added and the mixture incubated for 30 min at 37 °C in the dark. The reaction was stopped by the addition of 400 µl RPMI medium. CFSE-labeled EVs were washed and collected by ultracentrifugation as described above. For their detection in CSF, fluorescence was measured at Ex = 492 nm and Em = 517 nm.

### Biochemical Assays

The concentration of proinflammatory cytokines was determined in plasma samples and CSF using the following ELISA kits: Quantikine Rat TNF-α (RTA00), interleukin-6 (IL6; R6000B), and interleukin-1β (IL-1ß; RLB00) (R&D Systems, Wiesbaden, Germany). All analyses were performed according to the manufacturer’s instructions.

### Statistics

All analyses were performed by an independent investigator blinded to the experimental conditions. Data in figures were presented as mean values ± standard deviation (S.D.). Differences between the groups (HpX versus control and LPS versus control) within normally distributed data were subjected to one-way ANOVA with following post hoc Tukey test using SPSS v22.0 (SPSS IBM, Chicago, USA). Results were considered significant at *p* ≤ 0.05.

## Results

In order to simulate severe infection, rats were treated with an intravenous injection of lipopolysaccharide (LPS). Partial hepatectomy (HpX) was used to reflect a typical surgical intervention without an organ-specific deterioration (that means without significant changes in albumin and liver enzymes). The HpX model has been described in detail previously (Plaschke et al. 2018b).

**Fig. 2:**
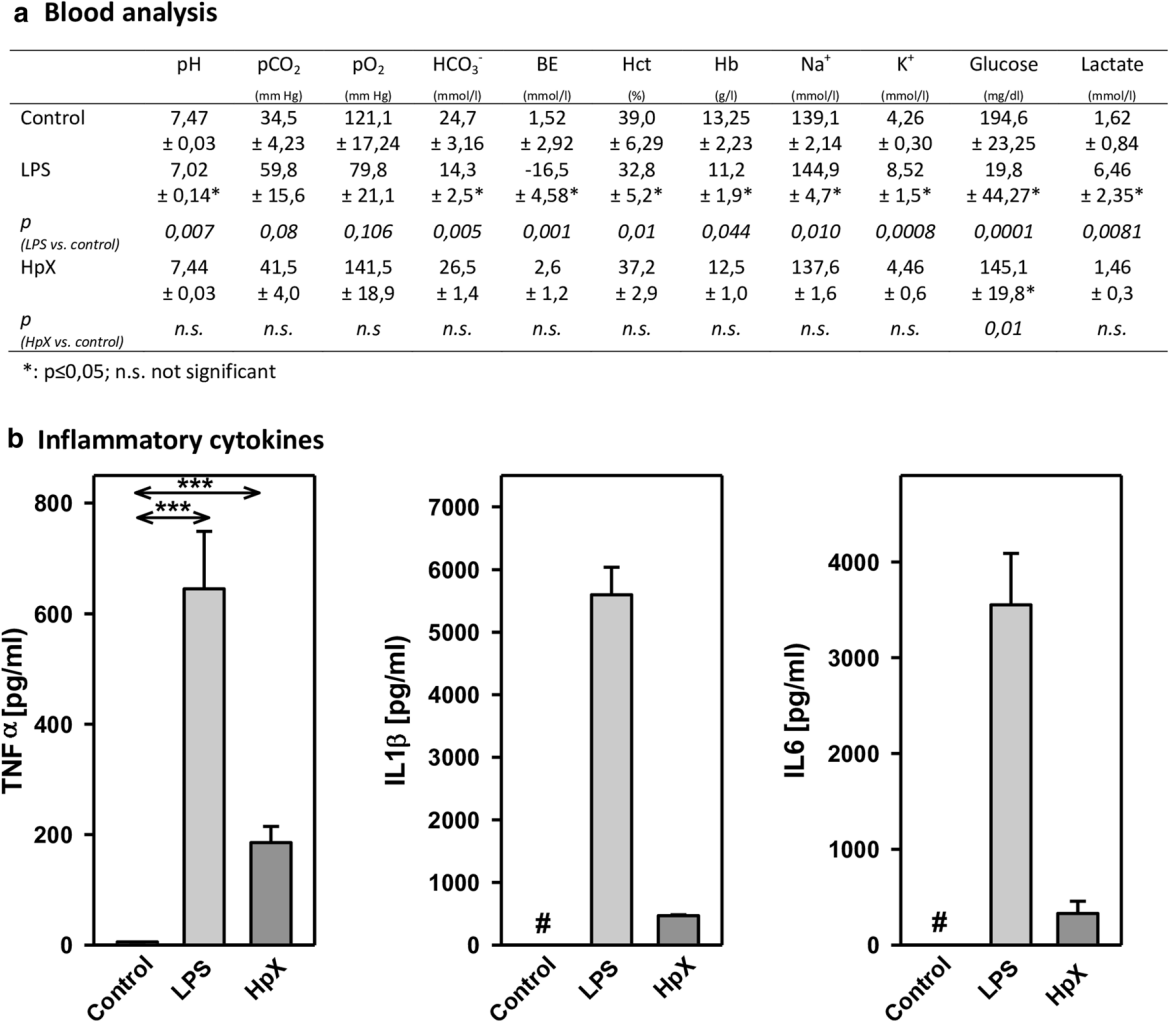
Blood parameters and cytokine release in LPS- and HpX-treated rats. A total of 18 adult male rats were randomly allocated to 3 groups with *n* = 6 per group. Control group was injected with 0.9% NaCl, LPS group received an injection of LPS (20 mg/kg body weight in 0.9% NaCl), HpX group was subjected to partial HpX. Blood was sampled 4 h after LPS and placebo injection, and 2 h after HpX. **a** Blood parameters were recorded at the end of the experimental period. *LPS* lipopolysaccharide, *HpX* partial hepatectomy, *BE* base excess, *Hct* hematocrit, *Hb* hemoglobin, *n.s.* not significant. *Significant effect (*p* < 0.05, one-way ANOVA with following post hoc Tukey test) between LPS or HpX versus control. **b** Inflammatory cytokines in the rats’ serum (obtained from the blood) at the end of the experimental period. Results are means of each group. #Below detection limit of the assay. Error bars indicate S.D. ***p < 0.001. In the case that values were below detection limit of the assay, no statistical analysis could be performed

Before administering LPS or performing HpX, no significant differences between the groups were observed for the following blood parameters: pH value, pCO_2_, pO_2_, bicarbonate (HCO_3_^−^), base excess (BE), hematocrit (Hct), hemoglobin (Hb), potassium (K^+^), sodium (Na^+^), glucose (Glu), and lactate (Lac) concentrations (data not shown). In untreated animals, all these measured parameters were in the physiological range. However, after treatment significant changes were found in pH value, K^+^, Na^+^, base excess, bicarbonate, glucose, and lactate concentration in endotoxemic (LPS-treated) rats compared to saline-injected control rats (*p ≤ 0.05, Fig. 2a)*.* Partial hepatectomy (HpX), however, did not induce significant changes in blood parameters, except for blood glucose concentration (Fig. 2a).

**Fig. 3:**
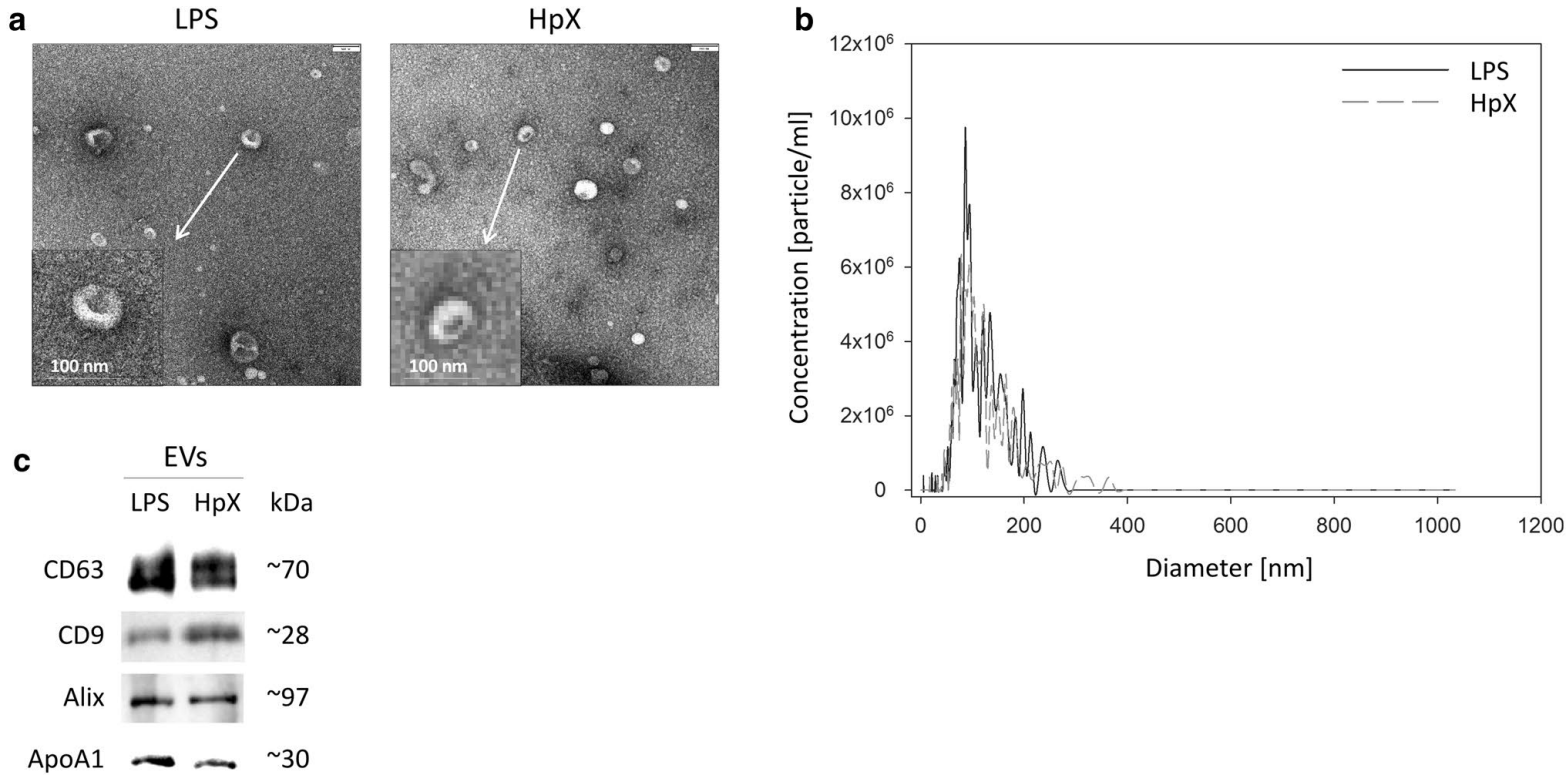
Characterization of rat serum-derived EVs. **a** Negative stain transmission electron microscopy revealed the size and shape of EVs concentrated from LPS- and HpX-treated rats. Scale bar 100 nm. **b** Nanoparticle tracking analysis determined the size distribution (*x-axis*) and particle concentration (*y-axis*) of the isolated yield. **c** Western blot analysis showed EV protein marker (CD63, CD9, Alix) and high-density lipoprotein marker (ApoA1) expression. Molecular weights (kDa) of each protein are indicated

**Fig. 4:**
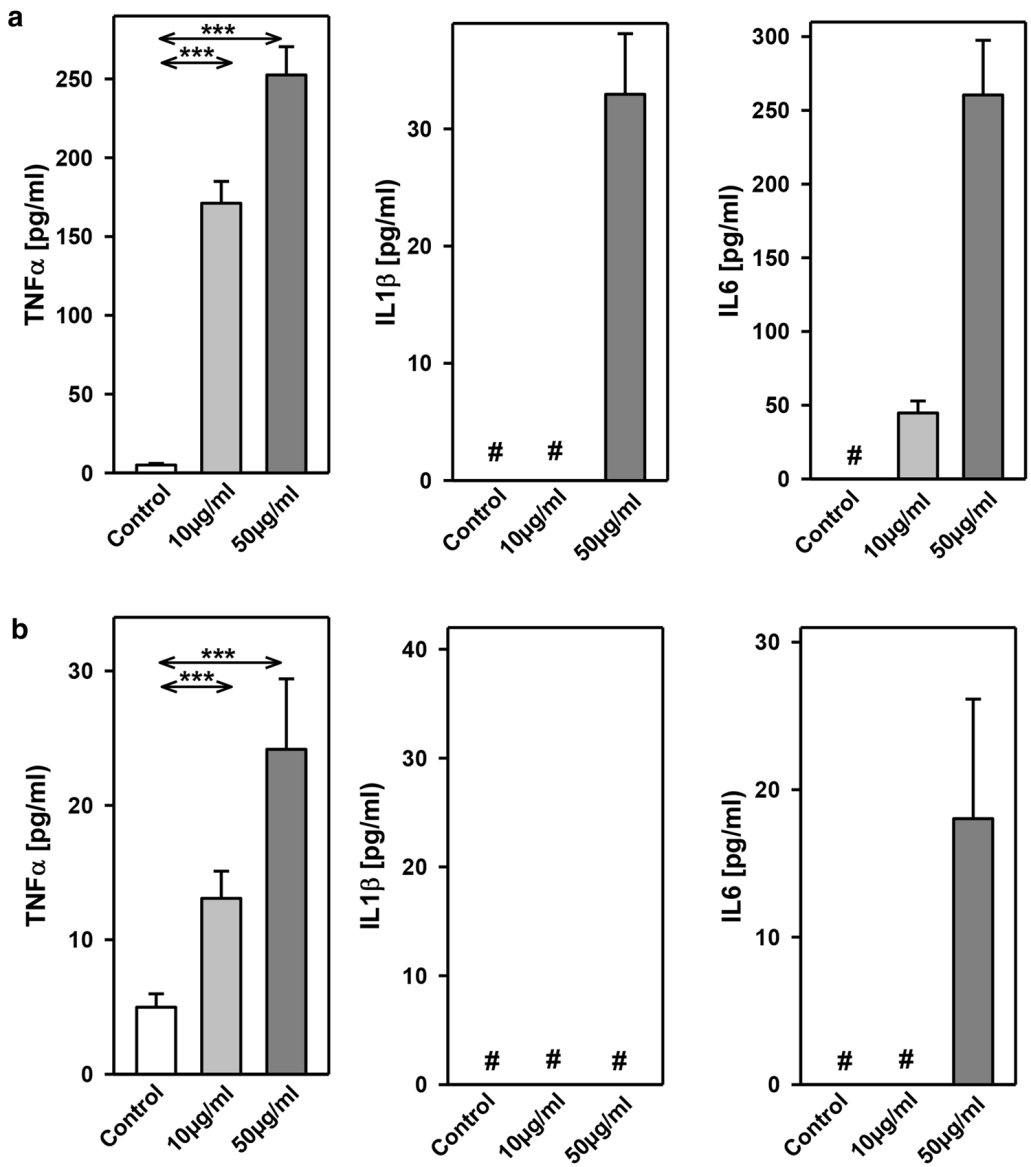
Effect of LPS- or HpX-derived EVs on cultured glia cells. EVs from LPS- (**a**) or HpX- (**b**) treated animals were added to the culture medium of HMC3 glia cells at the indicated concentrations. After 1 h the conditioned medium was assayed for TNF-α, IL1ß and IL6. Results are the means of 6 independent observations. Error bars indicate S.D. #Below detection limit of the assay. ***p < 0.001. In the case that values were below detection limit of the assay, no statistical analysis could be performed

**Fig. 5:**
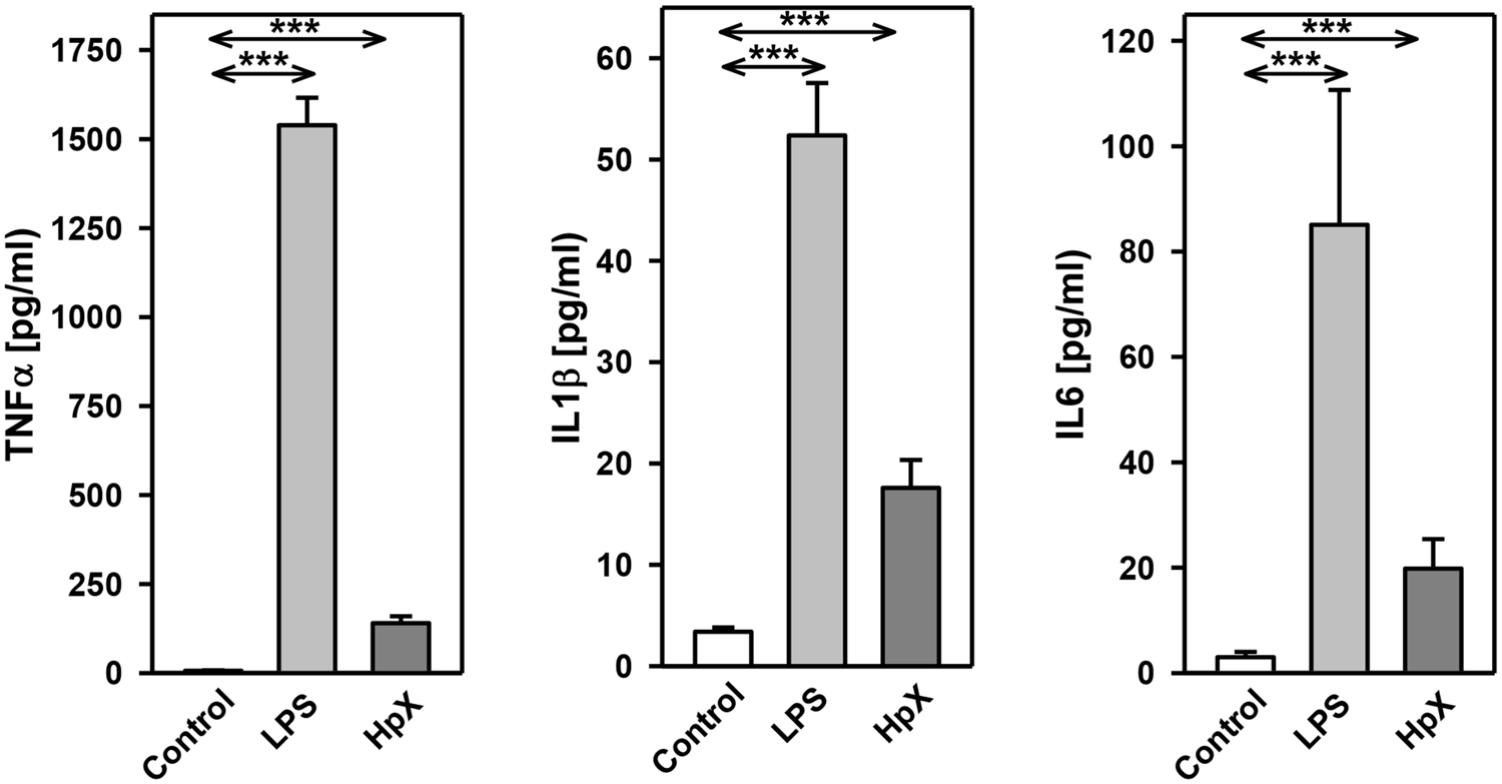
Cytokine release to the CSF after intracerebroventricular injection of LPS- or HPx-derived EVs. EVs (10 µg) from LPS- or HpX-treated animals were administered by intracerebroventricular (icv) injection to healthy rats. After 1 h CSF was taken and assayed for TNF-α, IL1ß and IL6. Results are the means of 6 independent observations. Error bars indicate S.D. ***p < 0.001

**Fig. 6:**
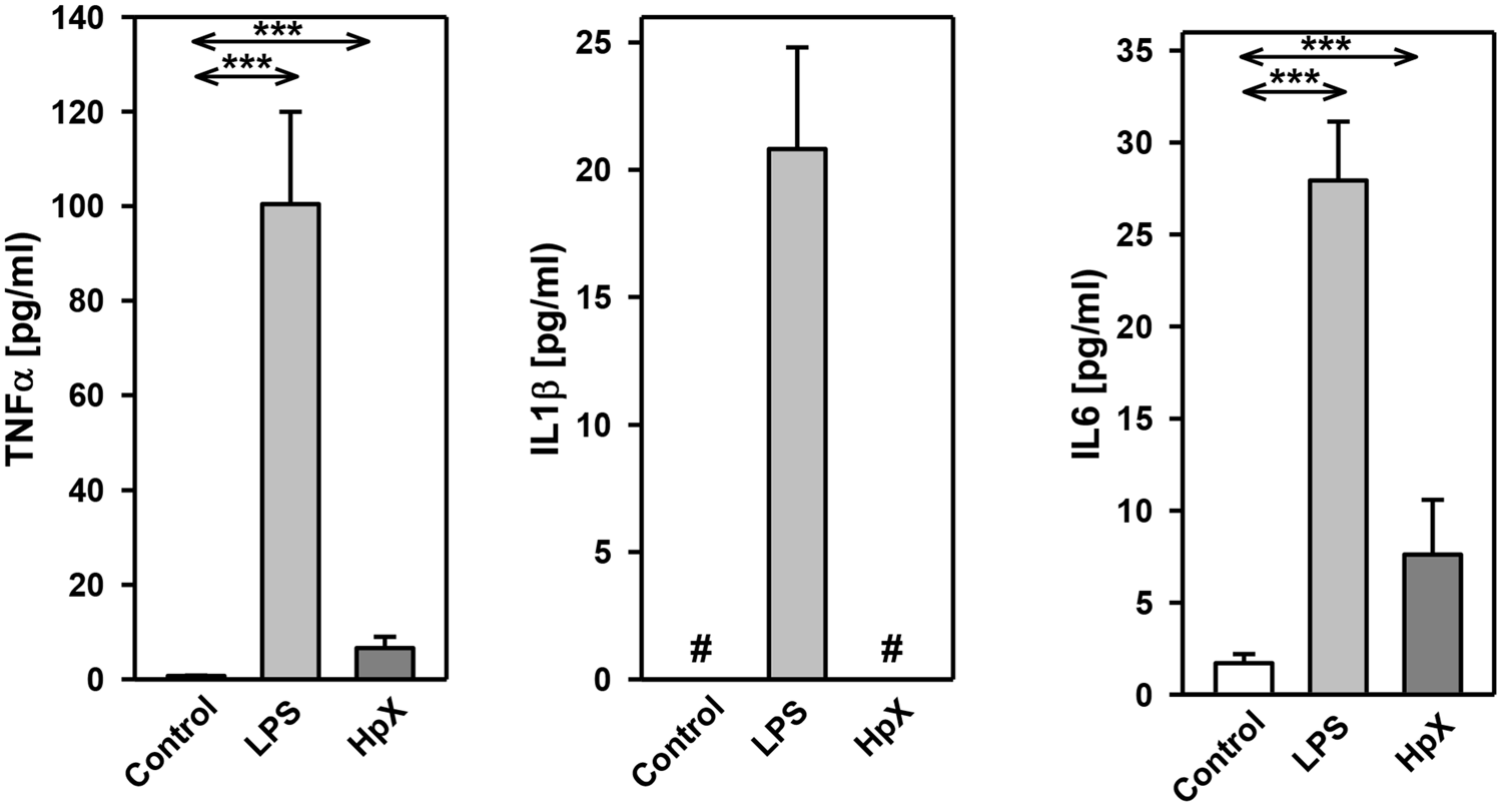
Cytokine release to the CSF after peripheral intravenous injection of LPS- or HpX-derived EVs. EVs (250 µg) from LPS- or HpX-treated animals were administered by intravenous injection via the femoral vene to healthy rats. After 1 h CSF was taken and assayed for TNF-α, IL1ß and IL6. Results are the means of 6 independent observations. Error bars indicate S.D. #Below detection limit of the assay. ***p < 0.001. In the case that values were below detection limit of the assay, no statistical analysis could be performed

The inflammatory cytokines TNF-α, interleukin-1ß (IL1ß) and interleukin-6 (IL6) were strikingly increased in LPS- and HpX-treated animals as compared to untreated controls indicating peripheral inflammation caused by endotoxemia or surgical treatment. The inflammatory reaction was more pronounced in the endotoxemic rats (Fig. 2b). EVs were then concentrated from both treatment groups (LPS, HpX) and untreated controls by differential ultracentrifugation and characterized by three different approaches (Thery et al. 2018). First, EV preparations were analyzed by negative stain transmission electron microscopy (TEM) to visualize the morphology and size of individual vesicles. As exemplified in Fig. 3a, lipid bilayer-enclosed particles that ranged in diameter from 30 to 150 nm were detected as round- and cup-shaped structures in all preparations. Second, nanoparticle tracking analysis (NTA) confirmed the concentration and size distribution of isolated particles. The majority of the yield ranged in a monopeak-fashion from 30 to 150 nm, resulting in mean diameters of 106.2 ± 43.4 nm (median 88.8 nm) for LPS samples or 109.0 ± 49.3 nm (median 91.7 nm) for HpX samples (Fig. 3b). Third, western blot (WB) analysis revealed the expression of three EV marker proteins in EV protein lysates (Fig. 3c). The two transmembrane proteins CD63 and CD9 as well as the cytosolic protein Alix were found to be expressed in EV protein lysates. Further, WB analysis also revealed the presence of apolipoprotein A1 (ApoA1) in all preparations (Fig. 3c), indicating that other extracellular particles, such as lipoproteins, were either co-sedimented or associated with EVs from rat serum samples. Taken together, our results demonstrate the successful enrichment of extracellular vesicles and particles from rat serum samples and no substantial difference was detected between all EV bulk preparations. The defined EV preparations were used for all further investigations. In order to test their direct effect on glia cells, HMC3 cells were treated with two concentrations of EVs (Fig. 1/sub-study I). EVs concentrated from LPS-treated animals strikingly induced the release of inflammatory cytokines from these cells (Fig. 4a). Likewise, EVs from HpX-treated animals also elicited the release of TNF-α and IL6, but at lower potency compared to EVs from LPS animals. No effect of HpX EVs on IL1ß secretion was detectable (Fig. 4b). Altogether, these results reflect the stronger inflammation in the LPS animals from which the EVs were isolated. In the next step, the in vivo action of the isolated EVs (LPS, HpX) was tested (Fig. 1/sub-study II). To this end, these EV preparations were injected intracerebroventricularly into the brain of healthy rats. LPS- and HpX-derived EVs induced the release of TNF-α, IL1ß and IL6 to the CSF (Fig. 5), indicating that proinflammatory EVs from the periphery have the potential to induce inflammation in the brain. This raised the question whether EVs in the periphery can pass the blood–brain barrier (BBB) thereby inducing neuroinflammation. To address this issue, we injected LPS- and HpX-induced EVs into a peripheral vein of rats and tested the induction of neuroinflammation by cytokine measurements in the CSF (Fig. 1/sub-study III). In fact, a striking increase of inflammatory cytokines in the CSF after peripheral injection of LPS-derived EVs indicates that they passed the BBB in amounts that can induce neuroinflammation (Fig. 6).

**Fig. 7:**
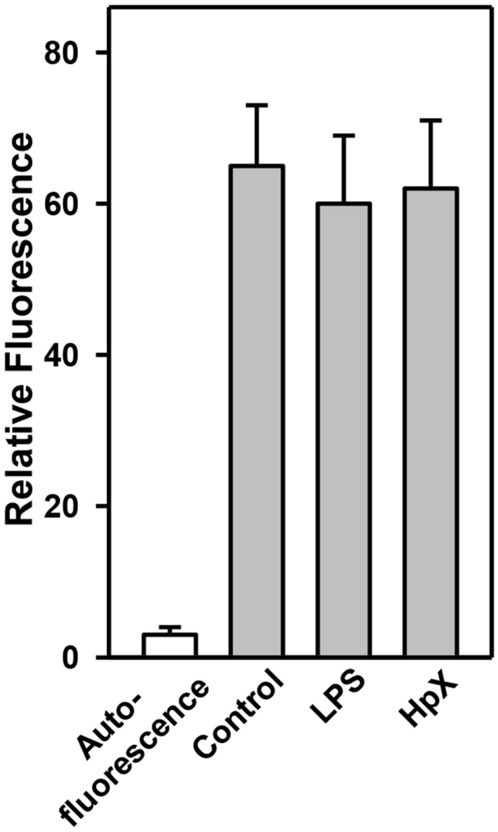
Detection of peripherally injected EVs in the CSF. EVs from healthy animals were fluorescein-labeled with CSFE and administered by intravenous injection via the femoral vein to LPS-, HpX-treated or control animals as described in Fig. 2. After 1 h CSF was taken and fluorescence measured. Results are the means of 3 measurements. Error bars indicate S.D

Final proof that peripherally injected EVs can reach the brain was obtained by peripheral intravenous injection of fluorescently labeled EVs and their detection in CSF. LPS-treatment or HpX had no effect on the permeability of the BBB for EVs (Fig. 7).

Since the anticholinergic drug physostigmine was suggested to attenuate neuroinflammation induced by peripheral inflammation, we also tested whether the administration of the drug to the animals from which the vesicles were concentrated can alleviate the action of the LPS- and HpX-derived EVs. An attenuating action of physostigmine was observed in all three sub-studies (Table 1).

**Table 1:** Effect of physostigmine

		Reduction of cytokine release		
		TNFα (%)	IL1β (%)	IL6 (%)
Exosome donors	LPS	26.5	20.9	26.9
	HpX	22.6	18.7	28.0
Sub-study I	LPS	26.2	22.2	23.7
	HpX	28.6	–	24.4
Sub-study II	LPS	29.6	18.8	22.9
	HpX	25.7	24.0	26.7
Sub-study III	LPS	21.2	22.3	29.7
	HpX	21.7	–	26.4

## Discussion

The LPS model of systemic inflammation that mimics many of the initial clinical features of sepsis is considered ideally suited to investigate the pathogenic effects of acute inflammation (Seemann et al. 2017). Likewise, the model of HpX is well appropriate to imitate a typical surgical intervention without an organ-specific deterioration (Plaschke et al. 2018b). Therefore, these models were used to collect EV pools that represent the two inflammatory states which were confirmed by blood analyses and the detection of strikingly increased proinflammatory cytokines in blood samples from the animals. As endotoxemia produced a considerably stronger inflammatory response as surgical stress, the two models not only represent two different causes of peripheral inflammation, but also mimic different levels of inflammation. Microglia regulates cytokines and inflammatory processes in the brain and the release of proinflammatory cytokines from these cells indicates the initiation of a neuroinflammatory reaction in the brain. In particular, the release of the proinflammatory cytokines TNF-α, IL1β and IL6 is considered a major trigger of the inflammatory process (Becher et al. 2017). The human microglial HMC3 cell line provides a well-suited tool to analyze the potential of EV populations to activate a proinflammatory program in glial cells, since this cell line displays very similar functional properties, in particular with regard to proinflammatory cytokine release, as primary microglia cells (Dello Russo et al. 2018). Our results clearly proof that EVs concentrated from LPS- or HpX-animals are potent proinflammatory activators of microglia cells albeit with different efficiencies. The same amount of LPS-derived EVs induced a significantly stronger response than HpX-derived EVs reflecting the different severity of inflammation in the donor animals. This may be explained either by the secretion of more potent proinflammatory EVs or just by a higher proportion of proinflammatory EVs in the total pool of EVs from the LPS-treated donors. Although our data suggested an EV-associated proinflammatory signaling that mediated the induction of neuroinflammation in both LPS-conditions (LPS, endotoxemia) and LPS-free conditions (HpX, peripheral surgical stress), we cannot rule out that residual LPS might contribute to the pronounced effects. However, it was reported that the half-life of LPS in the circulation of rodents is only about 2 to 4 min (Yao et al. 2016), making it very unlikely that residual LPS caused any observed effects. Further, using labeled LPS with radioactive-iodine (I-LPS), it was suggested that brain uptake of circulating I-LPS is so low that most effects of peripherally administered LPS are likely mediated through LPS receptors located outside of the BBB or triggered by other extracellular mediators (e.g., vesicles) (Banks and Robinson 2010).

Our results obtained by icv injection confirmed that LPS- or HpX-derived EVs are potent inducers of neuroinflammation in vivo. The findings of the cell culture experiments suggest that glial activation is also a major trigger in vivo. This is in line with a study where glial activation was assessed by immunohistochemistry in rats that were treated with EVs from endotoxemic donors (Li et al. 2018). However, a contribution of other cells, e.g., astrocytes, cannot be excluded. Moreover, it cannot be ruled out that non-vesicular protein aggregates, soluble proteins or lipoprotein particles accounted for the proinflammatory responses to some extent. Evidence demonstrated that established EV isolation techniques (e.g., ultracentrifugation, ultrafiltration, size exclusion chromatography, precipitation, immunoaffinity) are not able to completely separate EVs from non-EV material, including lipoproteins (Brennan et al. 2020; Karimi et al. 2018; Simonsen 2017; Sódar et al. 2016; Takov et al. 2019; Webber and Clayton 2013; Yuana et al. 2014). Thus, lipoprotein contamination became an increasingly recognized obstacle/problem/topic in the field of EV research (Simonsen 2017; Thery et al. 2018). Although the EV isolation method itself plays a crucial role, no consensus on an optimal purification method has been achieved yet. It has been suggested that functional outcomes are dependent on the purification method (Takov et al. 2019). Here, we used differential ultracentrifugation to concentrate EVs from rat serum samples. Although the EV pellet will invariably encompass non-EV material and contaminating factors that co-sediment alongside EVs, ultracentrifugation is considered to be the most commonly used EV enrichment method (Gardiner et al. 2016). When compared to size exclusion chromatography, ultracentrifugation can lead to higher purity of EVs isolated from rat blood samples (Takov et al. 2019). In our study, the high-density lipoprotein (HDL) marker protein ApoA1 was found to be present in all EV preparations in almost similar concentrations, thereby excluding any treatment-associated effects caused by the apolipoprotein itself. It has been reported that some EVs carry lipoproteins, making it difficult to discriminate between lipoprotein contamination and EV-associated lipoproteins (Karimi et al. 2018; Sódar et al. 2016; van Niel et al. 2015). Future studies are required to investigate any possible biological impact of lipoproteins and other potential mediators that might trigger the EV-mediated proinflammatory signaling. This could be addressed in further experiments by combining multiple isolation methods, leading to a complete/improved/more stringent separation of extracellular particles and vesicles and purer EV populations (Gardiner et al. 2016; Karimi et al. 2018; Simonsen 2017; Takov et al. 2019). However, significant losses of EVs and high variability between EV preparations can be expected due to long procedures involving multiple handling steps. Novel EV isolation tools are in development that might help to overcome these limitations in the future.

EVs have already been described as potent vehicles that easily pass the BBB and deliver signaling molecules to the CNS (Das et al. 2019). Therefore, also proinflammatory EVs that are released into the peripheral blood stream should be able to transfer proinflammatory signals to the brain. Our results clearly confirm that EVs—when injected into the peripheral blood stream—can cross the BBB and transfer proinflammatory signals to the CNS. In future studies the question, which cargo molecules of the EVs carry the information that is transferred to the CNS, should be addressed. Top candidates are EV-packed miRNAs or proteins, since they have already been described as key players in sepsis and inflammatory tissue injury (Park et al. 2019). In circulating EVs of septic shock patients significant changes in a panel of 65 miRNAs compared to those of healthy controls were found and the pathways potentially influenced by these miRNAs were largely correlated with inflammatory response, oxidative damage, and cell-cycle checkpoint (Real et al. 2018). EV-associated miR-21, miR-126, miR-146, miR-155 are examples of already confirmed regulators of inflammation in sepsis (Alexander et al. 2015; Chu et al. 2016; Pan et al. 2019; Song et al. 2017). Examples of proteins that represent EV-effective molecules in sepsis or systemic inflammation are caspase-3, SIP/S1PRS, neutrophil elastase (Genschmer et al. 2019; Moon et al. 2015; Park et al. 2018).

It has been shown that pharmacologic cholinesterase inhibition improves survival in experimental sepsis (Hofer et al. 2008). Accordingly the acetylcholinesterase inhibitor physostigmine attenuates neuroinflammation induced by surgical stress or endotoxemia (Kalb et al. 2013; Plaschke et al. 2018b, 2016; von Haefen et al. 2018). In our present study, physostigmine attenuated the pro-neuroinflammatory action of LPS- and HpX-derived EVs. Two different working points of physostigmine action can be envisioned. Activation of the nicotinic acetylcholine receptor alpha7 subunit is required for inflammatory activation of macrophages and thus, reduction of acetylcholine in the periphery by physostigmine may be responsible for the effect. On the other hand, cholinergic modulation of microglial activation (Shytle et al. 2004) has been shown to attenuate neuroinflammation that was primarily induced by peripheral inflammation (Terrando et al. 2015). Since peripheral inflammation as well as neuroinflammation is under the control of the vagus nerve-based inflammatory reflex both may be effective. Specific inhibition of cholinergic induction of a peripheral inflammation response reduced neuroinflammation in a surgical model, suggesting that the peripheral working point of physostigmine is involved in its anti-neuroinflammatory action (Kalb et al. 2013). Our results indicate that an effect of physostigmine on the EV cargo or the release of proinflammatory EVs in the periphery might at least partially contribute to its impact on neuroinflammation. In consequence, this would mean that peripheral cholinergic signaling influences EV-mediated signaling from the periphery to the CNS. This should be confirmed and detailed in further studies.

Altogether our present study clearly indicates that EVs released in endotoxemia or by surgical stress have the potential to induce a proinflammatory reaction in microglia cells resulting in a release of inflammatory cytokines to the CSF. Since EVs primed by peripheral inflammation can easily translocate to the brain by passing through the BBB, EV proinflammatory signaling from the periphery to the CNS appears effective in the induction of neuroinflammation in sepsis or surgical stress. Peripheral cholinergic signaling could be involved in the initiation of the EV-mediated proinflammatory signaling. Detailed analysis of the cargo of proinflammatory EVs in already ongoing studies will help to understand the underlying mechanisms.
