# Correction to: Proinflammatory Extracellular Vesicle-Mediated Signaling Contributes to the Induction of Neuroinflammation in Animal Models of Endotoxemia and Peripheral Surgical Stress

**DOI:** 10.1007/s10571-021-01112-4

**Published:** 2021-06-08

**Authors:** F. Fricke, J. Gebert, J. Kopitz, K. Plaschke

**Affiliations:** 1 Institute of Pathology, University Hospital Heidelberg, Im Neuenheimer Feld 224, 69120 Heidelberg, Germany; 2 Department of Anesthesiology, University Hospital Heidelberg, Im Neuenheimer Feld 110, 69120 Heidelberg, Germany

## Correction to: Cellular and Molecular Neurobiology https://doi.org/10.1007/s10571-020-00905-3

The article “Proinflammatory Extracellular Vesicle-Mediated Signaling Contributes to the Induction of Neuroinflammation in Animal Models of Endotoxemia and Peripheral Surgical Stress”, written by F. Fricke, J. Gebert, J. Kopitz and K. Plaschke, was originally published electronically on the publisher’s internet portal on 18 June 2020 without open access. With the author(s)’ decision to opt for Open Choice the copyright of the article changed on 27 May 2021 to © The Author(s) 2020 and the article is forthwith distributed under a Creative Commons Attribution 4.0 International License, which permits use, sharing, adaptation, distribution and reproduction in any medium or format, as long as you give appropriate credit to the original author(s) and the source, provide a link to the Creative Commons licence, and indicate if changes were made. The images or other third party material in this article are included in the article’s Creative Commons licence, unless indicated otherwise in a credit line to the material. If material is not included in the article’s Creative Commons licence and your intended use is not permitted by statutory regulation or exceeds the permitted use, you will need to obtain permission directly from the copyright holder. To view a copy of this licence, visit http://creativecommons.org/licenses/by/4.0.

